# 95332 Intestinal inflammation and altered gut microbiota associated with inflammatory bowel disease render mice susceptible to Clostridioides difficile colonization and infection

**DOI:** 10.1017/cts.2021.634

**Published:** 2021-03-31

**Authors:** Lisa Abernathy-Closedeline R. Barron, James M. George, Kimberly C. Vendrov, Peter D.R. Higgins, Ingrid L. Bergin, Vincent B. Young

**Affiliations:** University of Michigan

## Abstract

ABSTRACT IMPACT: Use of this novel murine model of inflammatory bowel disease (IBD) and C. difficile infection (CDI) will aid in developing new clinical approaches to predict, diagnose, and treat CDI in the IBD population. OBJECTIVES/GOALS: IBD is associated with intestinal inflammation and alterations of the gut microbiota, both of which can diminish colonization resistance to C. difficile. Here, we sought to determine if IBD is sufficient to render mice susceptible to C. difficile colonization and infection in the absence of other perturbations, such as antibiotic treatment. METHODS/STUDY POPULATION: C57BL/6 IL-10-/- mice were colonized with Helicobacter hepaticus to trigger colonic inflammation akin to human IBD. Control mice, not colonized with H. hepaticus, were pretreated with the antibiotic cefoperazone to render the gut microbiota susceptible to CDI. Mice were then gavaged with spores of the toxigenic C. difficile strain VPI 10463 and monitored for C. difficile colonization and disease. The fecal microbiota at the time of C. difficile exposure was profiled by 16S rRNA gene sequencing and analyzed using mothur. Statistical analyses were performed using R. RESULTS/ANTICIPATED RESULTS: Mice with IBD harbored significantly distinct intestinal microbial communities compared to non-IBD controls at the time of C. difficile spore exposure (14 days post-IBD trigger). Mice with IBD were susceptible to persistent C. difficile colonization, while genetically identical non-IBD controls were resistant to C. difficile colonization. Concomitant IBD and CDI was associated with significantly worse clinical and intestinal disease than unaccompanied IBD. DISCUSSION/SIGNIFICANCE OF FINDINGS: Patients with IBD who develop concurrent CDI experience increased morbidity and mortality. These studies in a novel mouse model of IBD and CDI emphasize the dual importance of host responses and alterations of the gut microbiota in susceptibility to C. difficile colonization and infection in the setting of IBD.

